# The meaning of boredom: Properly managing childhood boredom could lead to more fulfilling lives

**DOI:** 10.1038/s44319-024-00155-0

**Published:** 2024-05-13

**Authors:** Izumi Uehara, Yuji Ikegaya

**Affiliations:** 1 Institute for Education and Human Development, Department of Psychology, Ochanomizu University, Tokyo, Japan; 2 Graduate School of Pharmaceutial Sciences, The University of Tokyo, Tokyo, Japan

**Keywords:** Evolution & Ecology, Neuroscience

## Abstract

Boredom is a unique human emotion. Understanding its causes and consequences and how people learn to cope with it to develop new ideas and inspiration could help to alleviate its negative effects.

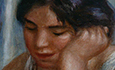

“The two enemies of human happiness are pain and boredom.” – Arthur Schopenhauer

Boredom is a unique human emotion that afflicts all of us at times: during a meeting or lecture, while waiting for an appointment, or in any other situation when we find ourselves with nothing meaningful to do. In Europe, the causes of boredom and its effect on the individual or society has long been an important topic of philosophy, science (Toohey, 2011) and the arts (Fig. 1) since the rise of modernity when industrialization enabled a wealthy middle class that liberated people from day-long labour.

**Figure 1:**
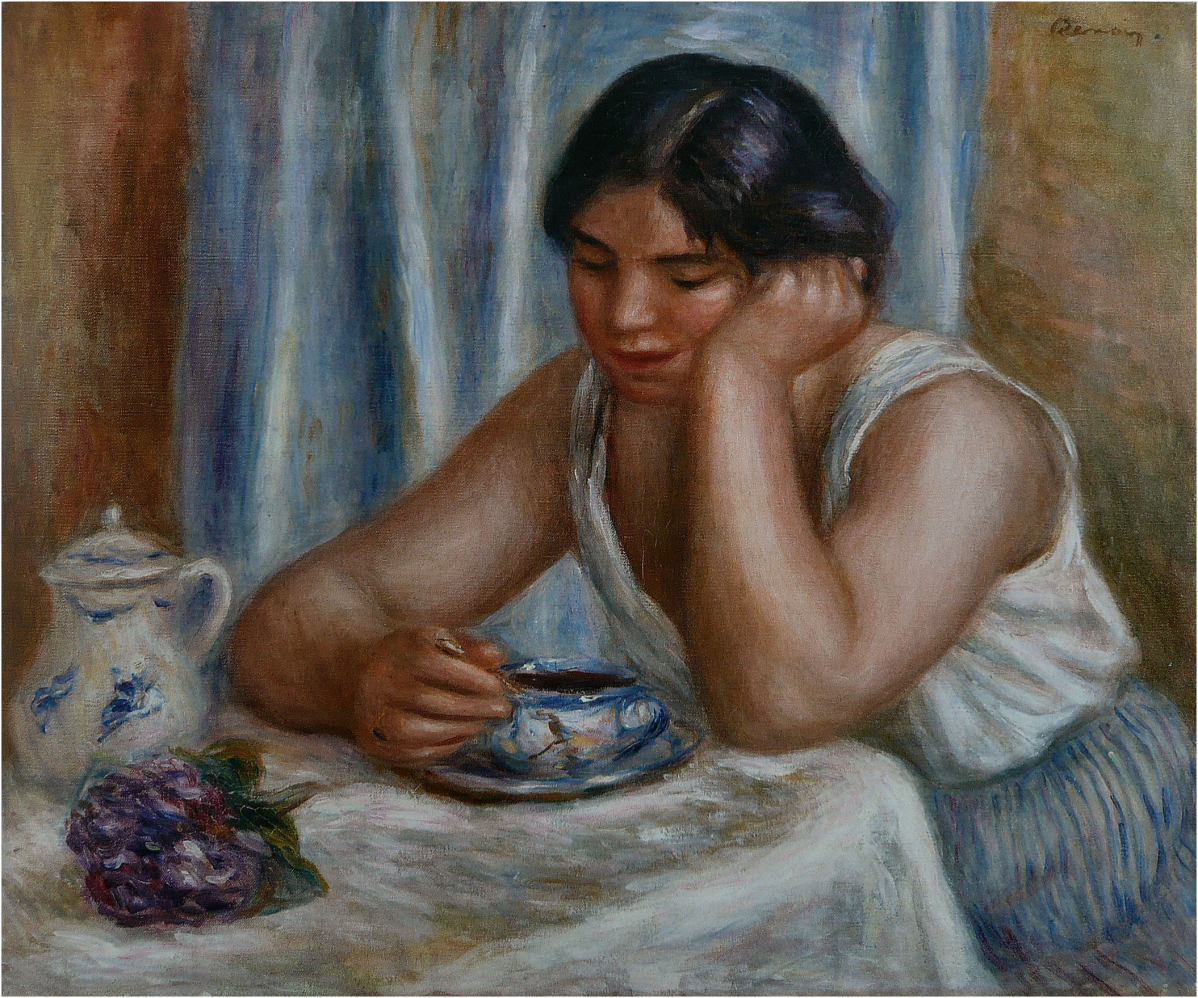
La tasse de chocolat (1912) by Pierre Auguste Renoir. Oil on Canvas. Wikimedia / Public Domain.

In the Western philosophical tradition, boredom attracted attention when the pursuit of ‘self’ and happiness came to be regarded as human rights. Many, such as Schopenhauer, have seen it as a negative emotion, a drag on happiness when we are forced to sit through a boring meeting, have to repeat the same task again and again, or spend time in an uninspiring environment. The German philosopher Friedrich Wilhelm Joseph Schelling said: “man seeks … driven by nature, to establish multiplicity and variety through rhythm. We cannot tolerate uniformity for very long, in everything that is in itself without meaning, for example in counting, we make periods.” (Bowie, 2018). Others, however, emphasized that boring has its positive side, too as idle time spent can inspire new ideas and inspiration. Friedrich Nietzsche, asserted that “‘For the thinker and for all inventive spirits, boredom is that unpleasant “doldrums” of the soul which precedes the happy journey and merry winds; he has to bear it, has to wait for its effect on him.”’ (Bowie, 2018).

“In the Western philosophical tradition, boredom attracted attention when the pursuit of ‘self’ and happiness came to be regarded as human rights.”

There are also cultural differences in our attitudes towards boredom. In Japan, for instance, people began to confront the notions of “self” and the pursuit of “happiness” rather late, even in the arts. As a consequence, boredom has not attracted much attention in science or philosophy—we are in the process of developing and validating a Japanese version of the Boredom Proneness Scale (BPS). Perhaps it is because of the long time before industrialization and modernity began to change social attitudes that people had to accept boredom as an ‘inevitability of life’ as much as storms or cold winters. Whatever the cultural differences and social attitudes towards it, academic research on boredom as a human emotion and trait is still scarce. In recent years, however, it has become evident from social-science and psychological studies that extreme boredom is associated with negative psychological symptoms and problematic behaviors such as internet addiction, meaningless violence or substance abuse.

## What is boredom?

But what is boredom? It can be generally defined as “the aversive experience of wanting, but being unable, to engage in satisfying activity” (Eastwood et al, 2012). It usually includes feelings of emptiness and meaninglessness, decreased motivation for action (Eastwood et al, 2012), and the inability to engage in any activity (Danckert and Merrifield, 2018). Boredom enjoys a rather negative reputation because of its prominent association with negative behaviors and symptoms. But it is also a fundamentally important emotion that we feel when we want to be absorbed in something or want to be busy but cannot. Moreover, it is related to an idle state of mind and mind wandering, which can turn into inspiration and novel ideas or become the driving force for meaningful activities (Danckert and Eastwood, 2020). The urge to engage in meaningful activities—in contrast to problematic activities—while feeling bored could lead to mental ‘fulfillment’ and well-being. If so, then boredom and learning how to cope with boredom in a meaningful and productive way may actually have beneficial effects both for the individual as well as for society.

“… boredom and learning how to cope with boredom in a meaningful and productive way may actually have beneficial effects both for the individual as well as for society.”

It is only in recent years that empirical studies of boredom have been conducted, such as psychological experiments to induce boredom in participants, recording brain activity associated with boredom, and medical investigations on its associations with negative symptoms and problematic behaviors. Most of these studies have relied on adolescents or adult participants, and we still know very little about the causes and effects of boredom in children or how they cope with it. This is a missed opportunity, since many adult behaviors, including how to deal with boredom, are learned during early childhood. A better understanding of boredom in toddlers and young children could, therefore, inform educational strategies or other interventions to alleviate its negative effects in children and later in adulthood.

## Two states of boredom

Boredom can be distinguished into two different aspects (Vodanovich and Watt, 2016). One is state boredom, the temporary experience of being bored in certain situations, such as having nothing to do in an uninspiring situation or when performing the same simple task over and over again. The other aspect is boredom as an innate trait, that is, the individual proneness to be bored (Farmer and Sundberg, 1986); indeed, people with a high innate propensity to experience boredom are more likely to fall into a state of boredom. Moreover, high boredom proneness has been associated with ADHD (Malkovsky et al, (2012)), risk-taking behaviors, a higher risk of dropping out of school, mental symptoms such as apathy, depression and anxiety, and substance abuse (Bench and Lench, 2013; LePera, 2011). Wilson et al, (2014) reported that several participants—especially men—when experimentally put into a situation of ultimate boredom for 6 to 15 min, preferred to administer electric shocks to themselves rather than having nothing to do and being left to themselves.

Given the various negative effects associated with a higher propensity to be bored has drawn more research attention to boredom proneness than to the effects of state boredom. However, it is not known yet if these negative symptoms and problematic behaviors are associated with or indeed caused by a higher innate propensity to become bored or if prolonged states of boredom can also cause these effects even among those who do not have a high boredom proneness. It would therefore be necessary to empirically investigate if prolonged states of boredom, such as working in a job characterized by doing the same task over longer times, can induce negative symptoms and behaviors irrespective of the innate propensity or if people with a lower propensity to fall into a state of boredom are better able to avoid such states so as to better comprehend the mechanism by which boredom arises.

## Children’s proneness to be bored

Boredom proneness is assumed to be innate to some degree, as it implies an individual’s susceptibility to boredom as a disposition. Certainly, there appear to be individual differences, but these do not necessarily all appear to be innate: anyone can be bored at any time. Among those who are more susceptible to boredom, some may engage in problematic behaviors, while others may be better able to cope with it and motivate themselves to conduct enjoyable and meaningful activities. To understand how innate the tendency to be bored is and to what extent one can cope with boredom and learn to cope with it, it would be useful to investigate boredom in childhood and its developmental processes.

Children are more easily bored, and their boredom is more pronounced than in adults. Young children are often unable to sit still, and it is still unknown how their remarkable restlessness and the tendency to be quickly bored develops. Although some studies have examined the relationship between schoolchildren’s levels of boredom in class and their grades, and how to decrease boredom in class (Jie et al, 2022; Tze et al, 2016), very few studies have investigated children’s boredom in their daily lives, such as situations in which children are likely to be bored, the behaviors they likely exhibit when bored, and how children learn to cope with boredom as they grow older. In fact, if children can learn to avoid boredom, they will be better able to cope with boredom, engage in enriching activities, and maintain mental health later in life. In this sense, accumulated studies on childhood boredom and its developmental processes are definitely needed.

“… if children can learn to avoid boredom, they will be better able to cope with boredom, engage in enriching activities, and maintain mental health later in life.”

Children’s susceptibility to boredom may be partly due to their innate disposition, but it is also owing to their restricted ability to cope with boredom. Our own study suggests that young children may not even be able to joyfully play for long periods of time at all (Ishibashi and Uehara, 2020). We studied the reactions of toddlers aged 15 to 35 months old when they were allowed to play with body-sized toys—slides, cars, and so on—and then changed the same toys to miniature size. Toddlers who treated the miniature-sized toys in the same way as full-sized toys—those who expressed so-called “scale errors”—became grumpy and bored, whereas the children who changed their play style tended to engage in pretend play with the miniature toys for longer. This observation suggests that the ability to flexibly change play styles in response to changing circumstances may play a role in whether or not children can continue to play with toys. This may also appear to be the case for coping with boredom. When boredom strikes, being interested in multiple aspects of life and being able to play flexibly and have fun may it easier for people to escape boredom and find fulfillment.

The greatest influence, both genetically (innately) and environmentally, on children’s behavior is likely to come from caregivers, especially the biological parents. This would also apply to children’s behavior when they are bored. Thus, we examined the relationship between parents, including their boredom tendencies and daily interactions, such as parenting behaviors, and various child behaviors, including their proneness to boredom. The results of this study are now in preparation for submission, and some of the findings were presented at a recent conference (Zhou et al, 2024), so we will summarize the results.

We conducted a questionnaire survey of more than 300 parents of early elementary school-aged children, primarily to explore the extent to which parents’ boredom proneness, ADHD tendencies, and parenting behavior influenced their children’s level of boredom and related ADHD tendency as a proxy for innate proneness to being easily bored. It is important to note that parenting behaviors are not the cause of ADHD or extreme boredom tendencies but may influence the degree of those symptoms. ADHD has been related to the dysfunction of specific brain networks and high heritability (Cortese and Coghill, 2018). Moreover, a combination of medication and behavioral therapy is effective in treating symptoms of ADHD, and one of the behavioral therapies involves parenting behavior.

The child’s ADHD tendencies were significantly correlated with parents’ ADHD tendencies, and the child’s, father’s and mother’s ADHD tendencies were significantly correlated with boredom tendencies. However, because the explanatory power of parents’ ADHD tendency and boredom tendency was low when the child’s ADHD tendency was extremely high, we examined other variables that would explain those tendencies. The results showed that the mother’s parenting responsiveness alone could explain a child’s extremely high ADHD tendency to a large extent, which suggests that high maternal responsiveness may induce strong ADHD-like behaviors in children. Overall, we showed a significant relationship between ADHD and boredom tendencies, not only within each parent and child, but also between parents and their children. Interestingly, although traditional parent–child studies have primarily examined influences from the mother, the father’s ADHD, and boredom tendencies were also shown to be related to those of their children. Furthermore, it was suggested that higher father responsiveness may contribute to children’s higher boredom tendency when children’s ADHD tendencies are high. Thus, we may say that our data indicate that parenting behavior can partially contribute to changing both ADHD and boredom symptoms in children for the better.

In summary, boredom, which, if well managed, can drive one to seek fulfilling activities and the acquisition of skills to better enjoy life, is undoubtedly influenced to some degree by caregivers, whether innate or from the nurturing environment. The findings from our studies of early parent–child relationships and parent–child boredom may be therefore useful for alleviating or even preventing problematic behaviors that result from extreme boredom, which are particularly pronounced in adolescence and beyond.

## The origins and mechanisms of boredom

In any case, many questions about boredom remain unanswered: To what extent is the tendency to be bored determined by genetic factors? Is boredom unique to humans? What are the evolutionary origins of boredom? What is the mechanism by which boredom is produced, not only mentally but also neurologically? To answer these, it is necessary to further examine the developmental mechanisms of boredom during childhood, to study whether boredom is observed in animals other than humans and to elucidate the neuroscientific mechanisms of boredom.

Human studies of the neural mechanisms of boredom have typically used functional magnetic resonance imaging. For example, a study in which participants watched a monotonous, low-salience video showed that the prefrontal, precuneus, and posterior cingulate cortex show correlated activity with boredom, while the insular cortex shows anticorrelated activity (Danckert and Merrifield, 2018). The prefrontal and insular cortices are also activated when participants perform a boring task in which they are asked to answer whether landscape images presented on a screen are blurred or not (Dal Mas and Wittmann, 2017).

More detailed neural mechanisms of boredom have been investigated primarily in animal studies. Although there has long been debate about whether or not animals can be bored, recent studies suggest that at least some aspects of human boredom appear as well. In a pioneer study, minks were housed in large cages either in an enriched environment with toys and obstacles or a dull, non-enriched environment. The frequency of behavioral contact with novel, even aversive, stimuli increased in the non-enriched group. Because this experimental design is equivalent to the way boredom has been quantified in humans, this behavioral change in animals is referred to as a “boredom-like state” (Meagher et al, 2017). We have also found that mice placed in a minimally stimulated chamber voluntarily receive aversive stimuli (Yawata et al, 2023). By recording or manipulating their neural activity, we found that activity of the insular cortex correlates with the production of boredom-like behavior, and boredom-like behavior is preceded by a transient release of dopamine in the ventral lateral striatum.

“Although there has long been debate about whether or not animals can be bored, recent studies suggest that at least some aspects of human boredom appear as well.”

The observation of boredom-like behaviors in animals may come as a surprise because boredom is not an emotion directly related to animal life. Although their boredom state is not necessarily the same as in humans, the behavior of seeking new stimuli—even repulsive stimuli—in the absence of other stimuli seems similar to the behavior of human children who are easily bored and mischievous. Another behavior found in animals relatively close to humans that is not directly related to survival is play. Animal play, like human play, is spontaneous and occurs in leisure time when not in danger or eating (Burghardt, 2005). Although this is speculative, play may attenuate the boredom state in both animals and children; more generally, meaningful, self-fulfilling behaviors associated with reducing chronic boredom, which everyone experiences to varying degrees as they age, may be regarded as an extension of play.

In any case, these findings indicate that insular cortical activities that are common to animals and humans may be involved in the neurophysiological mechanisms of boredom. By analyzing how the prefrontal cortex activity is involved in this mechanism, whether dopamine is released during boredom in humans, and the evolutionary relationship between these neurophysiological activities and behavioral traits, we may be able to better elucidate the neurological mechanisms that characterize a bored state.

## Summary

In summary, some mammals, such as mink and mice, have been shown to exhibit boredom-like behaviors similar to those of humans, so it is likely an evolved emotion rather than a cultural trait unique to humans. Moreover, findings in mice (Yawata et al, 2023) and our ongoing study of parents (Zhou et al, 2024) suggest that an individual’s boredom is not necessarily influenced solely by genetics, and the way how they cope with boredom may also be influenced by their upbringing. Although boredom is not directly a life-related emotion, such as appetite, fear, or sexual desire, but seemingly meaningless and futile, boredom may be involved in some life-ability or life-willingness, given that it is also observed in animals. The fact that boredom is an emotion that arises from not being able to find something to engage in, or from not being able to do what one wants to do, suggests that, especially in humans, the ability to cope with boredom itself may be related to fruitful living. For example, the ability to engage in hobbies and goal-directed behaviors would correspond to the ability to avoid boredom. Since the ability to cope with boredom is acquired during childhood, extensive research is needed on the developmental process of an individual’s boredom tendency and boredom-control skills from childhood to adolescence.

What skills are involved during childhood to develop the ability to cope with boredom? We suspect it is play and narrative. To being able to avoid boredom during childhood must be related to the extent to which a child is able to play by itself in a fun way for as long as possible. In addition, a lot of our conversations with fellow humans consist of telling stories and interesting episodes, so narrative abilities are important for our lives. Those narrative abilities are also acquired during childhood, and are related to thinking and imagination, which in turn lead to interest and motivation in many other things. In this sense, the developmental links between the “ability to play”, the “ability to tell stories” and the “ability to cope with boredom” could become topics of future research.

“… the developmental links between the “ability to play”, the “ability to tell stories” and the “ability to cope with boredom” could become topics of future research.”

## Can an AI be bored?

Emotion is a human cognitive function, and it would, therefore, be very difficult to simulate in an AI or AI-controlled robot; boredom may probably be the most difficult emotion to simulate at all. It may become possible to implement “happy” or “sad” emotions in silico, by making an AI or robot learn from happy and sad examples. But how would we “teach” an AI or robot to be bored? Boredom, which is a common emotion in daily life, can occur at many levels and in many situations, with greater individual differences than any other emotion. In an ultimately stimulation-free situation with “no sounds, no light, no movements possible”, how would an AI or robot react? Would it react with idle thoughts, imagination or ideas as a human would do to try to mentally escape this situation? Perhaps it could only show no reaction at all or shut down its processing.

“In an ultimately stimulation-free situation with ‘no sounds, no light, no movements possible’, how would an AI or robot react?”

For many reasons, it is necessary to study the developmental process of boredom, its effects on mental health, and the ability to control boredom, together with its cultural differences. This knowledge could inspire and inform educational strategies and other methods to help people better control boredom and improve their well-being. In addition, the study of boredom, which is one of the most unique emotions in humans and probably the most difficult for AIs and robots to truly emulate, may provide clues as to how humans should cope with the ongoing changes in society where AI and other advanced technologies are increasingly taking over mundane jobs and tasks from human labour. Just as the study of boredom became the topic of philosophical and scientific inquiries in the wake of modernity and industrialization, the ongoing digital revolution with the arrival of AI may spur further studies of the biological and neuronal mechanism of boredom, its evolution and development during childhood and its effects on society.

### Supplementary information

Peer Review File
