# Supplementary material for: The meaning of boredom: Properly managing childhood boredom could lead to more fulfilling lives
Source: EMBO Rep. 2024 May 13;25(6):2515–9. doi: 10.1038/s44319-024-00155-0 (PMC11169528; doi:10.1038/s44319-024-00155-0)
Supplement: Supplementary file 1 — Peer Review File [file 44319_2024_155_MOESM1_ESM.pdf]

EMBO reports

The meaning of boredom

Izumi Uehara and Yuji Ikegaya

Corresponding author(s): Izumi Uehara (uehara.izumi@ocha.ac.jp) , Yuji Ikegaya (yuji@ikegaya.jp)

**Review Timeline:**	Submission Date:	15th Apr 24
	Accepted:	26th Apr 24

Editor: Holger Breithaupt

Transaction Report: This article was editorially reviewed.
